# Host mechanisms involved in cattle *Escherichia coli* O157 shedding: a fundamental understanding for reducing foodborne pathogen in food animal production

**DOI:** 10.1038/s41598-017-06737-4

**Published:** 2017-08-09

**Authors:** Ou Wang, Tim A. McAllister, Graham Plastow, Kim Stanford, Brent Selinger, Le Luo Guan

**Affiliations:** 1 Department of Agricultural, Food and Nutritional Science, University of Alberta, Edmonton, AB T6G 2P5 Canada; 2 Agriculture and Agri-Food Canada, Lethbridge Research Centre, Lethbridge, AB T1J 4B1 Canada; 3 Alberta Agriculture and Forestry, Lethbridge, AB T1J 4V6 Canada; 4 Department of Biological Sciences, University of Lethbridge, Lethbridge, AB T1K 3M4 Canada

## Abstract

The host mechanisms involved in *Escherichia coli* O157 super-shedding in cattle is largely unknown. In this study, the comparison of transcriptomes of intestinal tissues between super-shedders (SS) and cattle negative for *E. coli* O157 (NS) was performed, aiming to identify genes that are potentially associated with super-shedding. In total, 16,846 ± 639 (cecum) to 18,137 ± 696 (distal jejunum) were expressed throughout the intestine, with the expressed genes associated with immune functions more pronounced in the small intestine. In total, 351 differentially expressed (DE) genes were identified throughout the intestine between SS and NS, with 101 being up-regulated and 250 down-regulated in SS. Functional analysis revealed DE genes were involved in increased T-cell responses and cholesterol absorption in the distal jejunum and descending colon, and decreased B-cell maturation in the distal jejunum of SS. RNA-Seq based SNP discovery revealed that the mutations in seven DE genes involved in leukocyte activation and cholesterol transportation were associated with *E. coli* O157 shedding. Our findings suggest that T-cell responses and cholesterol metabolism in the intestinal tract may be associated with super-shedding phenomenon, and the SNPs in the DE genes are possibly associated with the observed gene expression difference between SS and NS.

## Introduction

The sustainable agriculture production concept of “from farm gate to food plate” makes food safety an important component through the beef production chain. *Escherichia coli* O157:H7 (*E. coli* O157) is a zoonotic pathogen which produces Shiga toxins that can cause severe disease in humans including bloody diarrhoea, haemolytic uremic syndrome and in some circumstances even death^1^. Scallan *et al*. (2011) estimated that this pathogen is linked to more than sixty thousands cases of illness in humans and caused more than one hundred million dollars losses from 2000–2008 in United States annually^1^. Cattle are the primary reservoir for *E. coli* O157, and individuals that shed greater than 10^4^ CFU/g faeces are defined as super-shedders (SS)^2^. *E. coli* O157 originated from cattle can be spread into the farms and processing environments where it contaminates vegetables and beef products through the food production chain^3^. *E. coli* O157 can be shed by cattle for days, weeks or even months^4^, with shedding patterns differing substantially among individuals^2^.

Factors including environment, host, and microbes have been proposed to affect super-shedding^5^. Among these factors, the environmental factors including diet and seasonality have been reported to be associated with super-shedding^3, 6^. With regard to the microbial factors, *in vitro* studies have revealed that several *E. coli* O157 non-LEE (locus for enterocyte effacement)-encoded type III effector proteins may impair host innate immunity and that Shiga toxins can repress lymphocytes responses^7, 8^, which could lead to potential colonisation of this pathogen *in vivo*. It has been reported that a high challenge does of *E. coli* O157 to yearling steers it was able to cause damage to the epithelium of both the small and large intestine^4^. Also, *E. coli* O157 colonisation of the intestinal epithelium of challenged cattle could activate both innate and adaptive immune responses initiated by bovine macrophage and enterocytes through the recognition of the flagellum and lipopolysaccharide (LPS) of *E. coli* O157^8^. A recent study has also revealed distinct faecal microbiota between SS and cattle that are negative for *E. coli* O157 (non-shedders, NS)^9^. However, the host mechanisms involved in this foodborne pathogen shedding are largely unknown. Studies of the transcriptome of recto-anal junction (RAJ) of SS, the primary site of *E. coli* O157 colonisation^10^, revealed lower expression of genes involved in humoral and cell-mediated immune responses^11^. Based on the findings to date, super-shedding is a complicated process which may involve multi-biological processes and the mode of action regulating this process is unclear. Moreover, most attempts on understanding super-shedding were based on *E. coli* O157 challenged cattle^8^, and it is unknown whether the mechanisms of *E. coli* O157 shedding are the same between experimentally challenged and naturally-occurring SS.

It has been widely reported that less than 10% of the animals in a herd are SS^12–15^. However, it is difficult to explain such phenomena by only considering microbial (pathogen) and environmental factors, as all the animals that are fed with the same diet and raised in the same environment should have an equal possibility to ingest *E. coli* O157. In addition, the tropism *E. coli* O157 towards the RAJ^16–21^ of cattle cannot be fully explained by microbial effects solely. Therefore, we hypothesize that host gene expression throughout the whole gastrointestinal tract, as one of host related mechanisms, is different between SS and NS, and that the altered gene expression in SS, can be partially influenced by genetic variations. We also hypothesis that the difference in physiological functions determined by the transcriptome between small and the large intestine may contribute to the tropism of *E. coli* O157 colonisation in cattle. Therefore, the transcriptome profiles of tissues through the whole gastrointestinal tract including duodenum, proximal jejunum, distal jejunum, cecum, spiral colon and descending colon were compared between SS and NS using RNA-Seq and SNP discovery analysis was performed on the identified differentially expressed genes.

## Materials and Methods

The study was approved by the Animal Care Committee of the Lethbridge Research and Development Centre, Agriculture Agri-Food Canada (Animal Care Committee protocol number: 1120), and the steers were managed according to the Canadian Council of Animal Care Guidelines.

### Super-shedders identification and intestinal tissues collection

Super-shedder identification and the tissue sampling procedure was outlined in previous studies^11, 22^. Briefly, 50 g of faecal samples were collected from 400 yearling steers with similar body weights (452 kg ± 23 kg, mean ± standard deviations) that were on a finishing diet and were used for *E. coli* O157 enumeration using CT-SMAC agar (Dalynn Biologicals, Calgary, AB. Canada). Confirmation of O157 serogroup was performed using an *E. coli* O157 Latex Test kit (Oxoid Ltd, Basingstoke, Hampshire, UK). Isolates were further confirmed to be *E. coli* O157:H7 using a multiplex PCR assay targeting *VT*, *eaeA*, *fliC* ^23^. Steers identified with ≥10^4^ CFU of *E. coli* O157/g faeces were defined as SS. Of the 400 steers, 5 SS (out of 11 identified SS) and 5 NS pen-mates were transferred to the Lethbridge Research Centre research feedlot. Steers were slaughtered within 4–11 days after purchase, and all the SS were positive for *E. coli* O157 prior to tissue sampling although not all were shedding at >10^4^ CFU/g faeces^11^. Tissues of duodenum, proximal jejunum, distal jejunum, cecum, spiral colon and descending colon were collected immediately after slaughter and snap frozen in liquid nitrogen.

### RNA-extraction, RNA-Seq library preparation and RNA sequencing

RNA extraction and library preparation procedures have been previously outlined by Wang *et al*.^11^. Briefly, total RNA was immediately extracted from about 100 mg of ground powdered tissue using a mirVana total RNA Isolation Kit (Ambion, Carlsbad, CA, USA). One micro gram of RNA was used for library preparation using a Truseq Stranded Total RNA Sample Preparation kit (Illumina, San Diego, CA, USA) following the manufacturer’s instructions. The cDNAs were confirmed to meet quality control standards and sequenced (paired-end, 2 × 100 bp) using a HiSeq. 2000 sequencing system (Illumina, San Diego, CA, USA) at Genome Quebec Innovation Centre, Montreal, Quebec, Canada.

### Identification of transcriptomes and differentially expressed genes

The RNA-Seq data analysis was performed using the pipeline previously reported^11^. Briefly, adapter sequence removal and quality filtering of sequencing reads were performed by fastq-mcf^24^, and the remaining reads were mapped against the reference bovine genome UMD3.1 assembly^25^ using the splice junction mapper, Tophat2^26^. HtSeq-count^27^ was used to quantify the mapped reads to each known bovine gene. The number of mapped reads was then normalized into counts per million (cpm) to eliminate variation introduced by sequencing depth, using the formula: cpm = (Number of reads mapped to a gene) ÷ (total number of reads mapped to all annotated genes) × 10^6^. Any gene with a cpm higher than 1 was considered as being expressed^28^, and the genes expressed in more than 2 of animals in each group were further subjected to differential expression and functional analysis. Bioconductor package edgeR was used for differentially expressed (DE) gene identification between SS and NS in each intestinal tissue^29^, using negative binomially distributed RNA-Seq counts. A false discovery rate (FDR) of 0.05 was used as the cut-off to define DE genes. Log2 fold change of each DE gene was calculated using the equation: log2 fold change = log2(average cpm of SS/average cpm of NS). Log2 fold change threshold for DE genes were set to ≤−1 and ≥1 with negative values indicating down-regulated genes in SS and positive values indicating up-regulated in SS.

### Functional analysis of transcriptome and differentially expressed genes

Functional analysis of RNA-Seq data was performed using several bioinformatics tools. The PANTHER (Protein Analysis Through Evolutionary Relationships) classification system^30^ was used for gene ontology (GO) terms annotation and enrichment, with p-value 0.05 used as cut-off. Ingenuity Pathway Analysis® (IPA, QIAGEN, Redwood City, CA, United States www.qiagen.com/ingenuity) was used for functional term enrichment and canonical pathways analysis for core transcriptomes and DE genes for each tissue, and a downstream effects analysis was performed to identify the biological influence of the DE genes. As the maximal number of genes that IPA can analyze is 8,000, the 8,000 genes in the core transcriptome with the highest cpm values were used for functional analysis for core transcriptome of each tissue. Based on the fold change of DE genes, the IPA also predicted if a certain enriched biological function was increased or decreased, and a z-score was used to indicate whether a predicted function was increased (z-score >2.0) or decreased (z-score <2.0). A similar z-score algorithm was used by IPA to predict if a canonical pathway was inhibited (z-score <−2.0) or activated (z-score >2.0).

### RNA-Seq based SNP discovery

To identify potential causal effects of DE genes, single nucleotide polymorphism (SNP) analysis was performed using RNA-Seq reads for these DE genes. To increase read coverage for SNP analysis, RNA-Seq reads of all tissues were combined for each animal and SNP calling was performed by VarScan2^31^, which is a mutation caller that uses a heuristic algorithm for sequence variant detection. For SNP calling, the minimum base quality of reads was 15, minimum read depth at a position to call a SNP was 8, minimal read number that supports an allele was 2, and the minimum variant allele frequency threshold was 0.1. The SNPs were kept for association analysis by the Fisher’s exact test if the genotypes for all 10 animals were detected by VarScan2. Shedding of *E. coli* O157 was considered as a phenotype of SS, and the Fisher’s exact test was used to analyze for associations between alleles and super-shedding with significance at 0.05.

### Cholesterol quantitation

To quantify the cholesterol concentration in distal jejunum and descending colon, a Cholesterol Quantitation Kit (Catalog Number: MAK043, Sigma-Aldrich, St. Luis, MO, USA) was used for lipid extraction and quantitation by fluorometric detection following manufacturer’s instruction. Prior to cholesterol extraction, adipose tissues were removed manually using a scalpel, as the study of lipid storage was not the objective of this study, and the rest of the tissues were ground and mixed. Approximately 100 mg of tissue was used for cholesterol extraction and measurement according to the kit instructions, and the fluorescence intensity was detected at: λ_ex_ = 535 and λ_em_ = 587 nm. Two tailed sample t.test (significance level: 0.05) was performed to determine whether cholesterol content of the distal jejunum and descending colon differed between SS and NS.

### Availability of sequencing data

The RNA-Seq data are available at NCBI Gene Expression Omnibus (GEO) database under accession number GSE85277.

## Results

### Transcriptome profiling of bovine intestinal tissues

**Figure 1:**
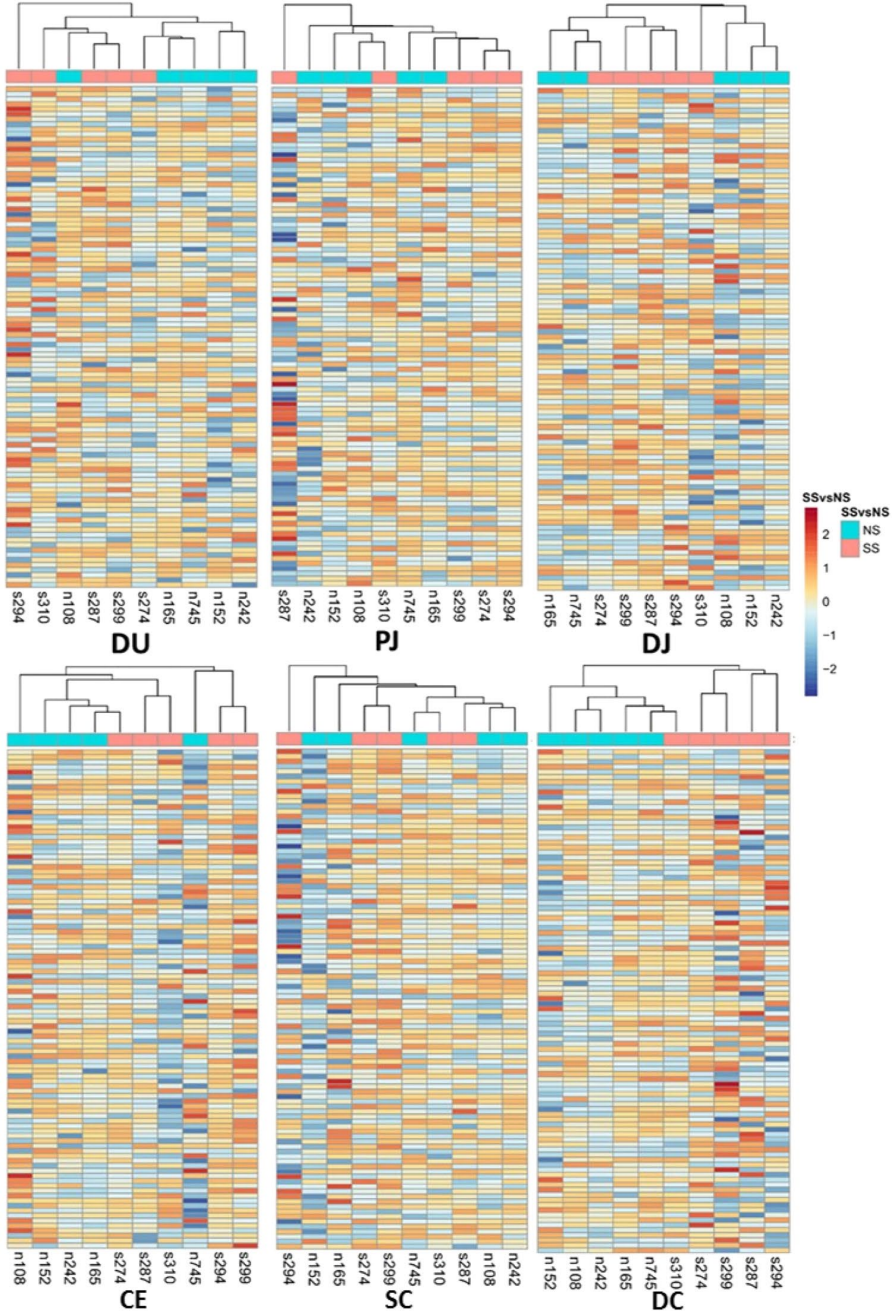
Heatmaps for core transcriptome of all tissues. The genes in core transcriptomes were first aggregated into 100 clusters using k-means clustering. DU, duodenum; PJ, proximal jejunum; DJ, distal jejunum; CE, cecum; SC, spiral colon; DC, descending colon.

**Figure 2:**
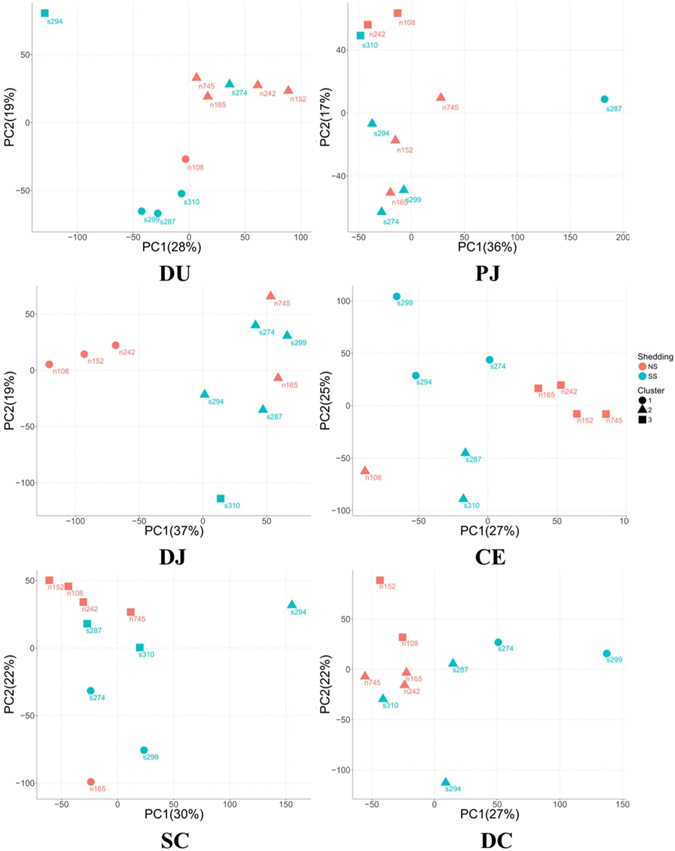
Principle component analysis for core transcriptome of all tissues. The red circles indicate non-shedders that are clustered together based on their gene expression profiles. The expression values of each gene were scaled by subtracting mean expression value then dividing by standard deviation. The cluster analysis was performed using k-mean algorithm. DU, duodenum; PJ, proximal jejunum; DJ, distal jejunum; CE, cecum; SC, spiral colon; DC, descending colon.

The number of paired-end sequence reads for each tissue sample ranged from 185.0 million in the descending colon to 203.7 million in the cecum (Supplementary Fig. S1A), with the number of the expressed genes detected in each tissue ranging from 16,846 ± 639 (mean ± standard deviation) in the cecum to 18,137 ± 696 (mean ± standard deviation) in the distal jejunum (Supplementary Fig. S1B). For each tissue, transcripts with counts per million higher than 1 (cpm ≥ 1) in all 10 animals were defined as the core transcriptome for that tissue. The core transcriptomes consisted of 12,813 expressed genes in the duodenum, 12,552 expressed genes in the proximal jejunum, 12,905 expressed genes in the distal jejunum, 12,627 expressed genes in the cecum, 12,404 expressed genes in the spiral colon, and 12,216 expressed genes in the descending colon (Supplementary Fig. S1C). The hierarchical cluster analysis of core transcriptomes revealed no clear separation between SS and NS in most of the gut regions, except for descending colon where SS (with the exception of SS 310) and NS were clustered into separate groups (Fig. 1). Principal component analysis (PCA) plots based on the whole transcriptome revealed that four of the NS were clustered closely for duodenum, cecum and spiral colon, three of NS were clustered closely for distal jejunum, and NS and SS were separated on the direction of first principal component (except for SS 310) for descending colon (Fig. 2). For SS animals, they were not closely grouped, with certain SS more closely related to NS in several tissues. For example, the duodenum transcriptome of SS 274 was similar to that of NS, while the spiral colon transcriptome of SS 287 and descending colon transcriptome of SS 310 were similar to those of NS.

### Functional analysis of core transcriptomes of bovine gastrointestinal tissues

**Figure 3:**
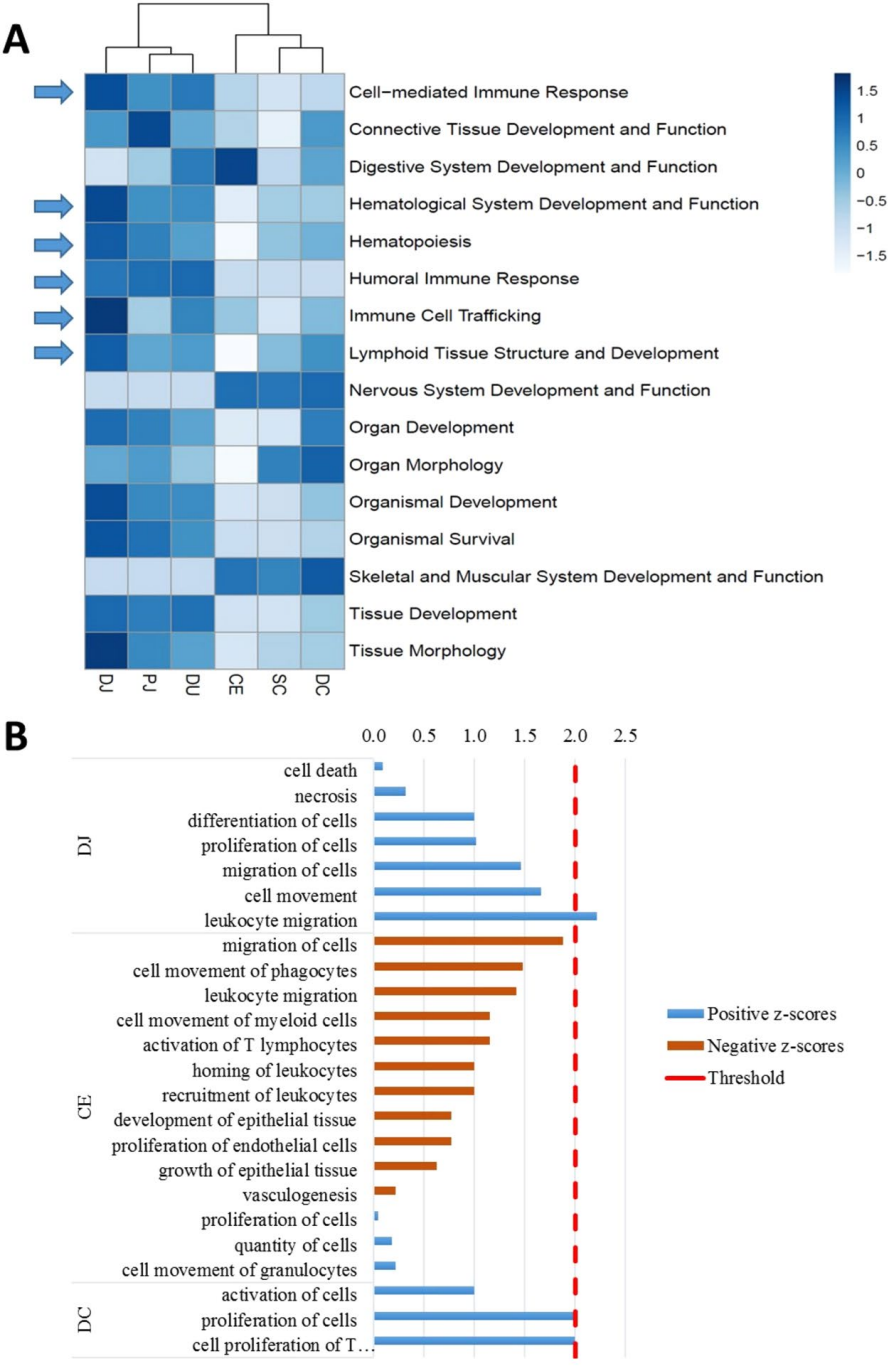
Function analysis for core transcriptome of cattle intestinal tissues. (**A**) IPA (Ingenuity Pathway Analysis) functional analysis of the core transcriptome of intestinal tissues. The heatmap shows scaled values of −log_10_(p-value) indicated with blue colours: the darker the blue, the more likelihood that a function is associated with the core transcriptome of a tissue. (**B**) Functional analysis for differentially expressed (DE) genes. Results obtained from IPA downstream analysis for DE genes of each tissue. Positive z-scores were indicated with blue bars and negative z-scores with brown bars. The absolute values of z-scores were plotted, and those with a threshold greater than 2 hr, considered to indicate a difference in functionality between super-shedders and non-shedders. A positive z-score indicates enhancement, while a negative z-score indicates a reduction in function. DJ, distal jejunum; CE, cecum; SC, spiral colon; DC, descending colon; for differentially expressed genes identified in the other tissues, no IPA functional terms were enriched.

Functional analysis of the core transcriptome using PANTHER for biological process GO terms (Gene Ontology) enrichment showed consistent enriched GO terms for all intestinal tissues, including metabolic processes, cellular processes, biological regulation, localization and development processes (based on p-values) (Supplementary Fig. S2). Further functional analysis using IPA revealed 16 enriched biological process categories (Fig. 3A). Although similar functional categories were identified for all intestinal tissues, the likelihood of association (indicated by Fisher’s exact test p-value calculated by IPA) between functional categories and the transcriptome of a tissue varied, rendering the tissues of small and large intestines clustered separately (Fig. 3A). Immune functions such as cell mediated immune responses, haematological system development and function, humoral immune responses and lymphoid tissue structure and development were more associated with the core transcriptome of small intestinal tissues than with those of large intestinal tissues, particularly the distal jejunum (Fig. 3A). Functional analysis by the Panther Classification System also showed that more transcripts (t.test, p-value < 0.05) in the core transcriptome of small intestinal tissues were associated with immune system processes: 562 transcripts in the duodenum, 574 transcripts in the proximal jejunum, 579 transcripts in the distal jejunum, 545 transcripts in the cecum, 497 genes in the spiral colon, and 514 transcripts in the descending colon.

### Identification of tissue dependent differentially expressed genes between super-shedders and non-shedders

**Figure 4:**
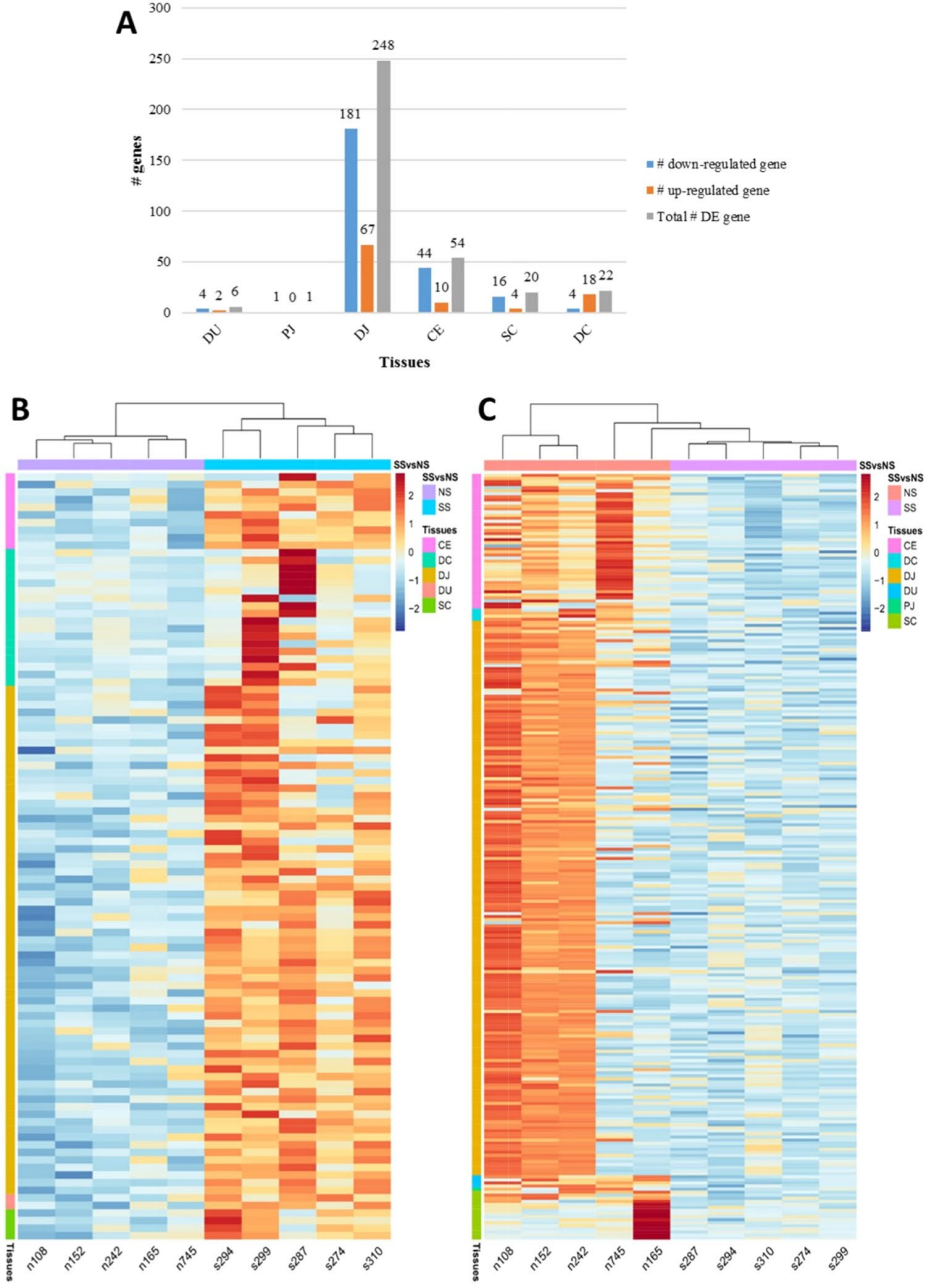
Differentially expressed genes between super-shedders and non-shedders. (**A**) Number of differentially expressed (DE) genes identified in intestinal regions. DE genes: FDR < 0.05, log2(fold change) <−1 or >1, cpm ≥1 in at least 50% of SS and NS. (**B**) and (**C**) are Heatmaps for log_2_(counts per million) of differentially expressed genes in all tissues: (**B**) up-regulated differentially expressed genes in super-shedders; (**C**) down-regulated differentially expressed genes in super-shedders. Scaled log2(counts per million) was indicated by red and blue colors, and red indicates higher expression level, while blue colour indicates lower expression level. The column labels indicate super-shedders and non-shedders: NS, non-shedders; SS, super-shedders. The row labels indicate the tissue where differentially expressed genes were identified. DU, duodenum; PJ, proximal jejunum; DJ, distal jejunum; CE, cecum; SC, spiral colon; DC, descending colon.

The number of differentially expressed (DE) genes ranged from 1 (in the proximal jejunum) to 248 in the distal jejunum (Fig. 4A) between SS and NS, and the Log2 (fold change) of DE genes ranged from 8.9 for *APOB* in the distal colon to −7.9 for *CPS1* in the spiral colon (Supplementary Table S1) of SS. In total, 101 genes were up-regulated and 250 were down-regulated when the transcriptomes of gut tissues of SS to NS were compared (Fig. 4A and B).

### Functional analysis of differentially expressed genes

Functional analysis using PANTHER revealed that the GO terms were enriched for distal jejunum and descending colon DE genes, but not for the duodenum, proximal jejunum, cecum or spiral colon DE genes (p-value > 0.05). Similarly, the IPA functional terms were enriched for DE genes identified from distal jejunum, cecum and descending colon, but not for those from duodenum, proximal jejunum, or the spiral colon.

For 67 up-regulated DE genes in distal jejunum, 33 enriched GO terms were identified within 11 functional classes based on the hierarchical structure of GO term (Supplementary Table S2). The most specific GO term in each enriched class includes “regulation of interferon-gamma production”, “positive regulation of inflammatory response” and “immune response-activating cell surface receptor signalling pathway” (Table 1). Among these 11 GO terms, 10 of them were associated with immune functions (Table 1), which involved 22 DE genes including five genes encoding cytokines (*CCL2*, *CXCL10*, *CXCL9*, *IL1A* and *TNFSF10*) and three genes encoding cytokine receptors (*IL18R1*, *IL1RL1* and *CCR9*) (Table 2). According to IPA functional analysis of DE genes of distal jejunum, five up-regulated genes (*F3*, *CCL2, CCR9, GPR132, S1PR2*) were associated with increased leukocyte migration (z-score ≥ 2.0) (Fig. 3B and Table 3), while five down-regulated DE genes (*BCL6, BLNK, CD79A, CD79B, EBF1*) were involved in B-cell signalling pathway (z-score ≤ −2.0) (Table 3, Supplementary Fig. S3). For DE genes in descending colon, IPA analysis showed four up-regulated genes (*APOA1*, *CD36*, *GP2* and *GPAM*) were associated with increased proliferation of T-cell function (z-score ≥ 2.0) in SS (Fig. 3B and Table 3).

**Table 1:** Gene ontology (GO) term enrichment (biological process, p-value < 0.05) for DE genes between non-shedders and super-shedders in distal jejunum and descending colon, no functional terms were enriched for other tissues (p-value ≥ 0.05).

GO Terms	Fold enrichment***	Genes
Up-regulated genes in DJ*		
regulation of interferon-gamma production	22.6	*LOC100848575, IL1RL1, PD-L1, KLRK1, IL18R1*
positive regulation of inflammatory response	22.6	*IL1RL1, CD6, IDO1, CCL2, GBP5*
immune response-activating cell surface receptor signaling pathway	22.5	*LOC100848575, PTPN22, IKT, THEMIS, ICP2, KLRK1*
negative regulation of homotypic cell-cell adhesion	22.0	*PTPN22, ADAMTS18, ALOX12, PD-L1, IDO1*
positive regulation of cytokine production	12.2	*LOC100848575, PTPN22, IL1RL1, IL1A, PD-L1, CD6, KLRK1, IL18R1, IDO1, GBP5*
negative regulation of immune system process	9.5	*PTPN22, IL1RL1, GPR171, PD-L1, KLRK1, IDO1, CCL2, GPR55*
cytokine-mediated signaling pathway	8.8	*CXCL10, IL1RL1, IL1A, CXCL9, CCR9, IL18R1, F3, CCL2*
leukocyte activation	8.4	*LOC100848575, PTPN22, BATF2, LCP2, ITK, CD6, KLRK1, IL18R1, THEMIS*
immune response	8.1	*BOLA-NC1, CLNK, CXCL10, TNFSF10, IL1A, LCP2, ITK, PD-L1, CD6, CXCL9, CCR9, CD1D, IL18R1, THEMIS, CCL2, LOC407111, GBP5, F1N3I2*
positive regulation of intracellular signal transduction	5.4	*CXCL10, TNFSF10, PTPN22, IL1A, CXCL9, IL8R1, F3, NRG1, CCL2, PPR55, LOC407111*
defense response	5.0	*CXCL10, BATF2, IL1A, ITK, CD6, CXCL9, KLRK1, F3, IDO1, CCL2, GBP5, IFI47*
**Down-regulated genes in DJ**		
regulation of nuclease activity	33.8	*HMGB1, NEIL1, PCNA, LOC618297*
base-excision repair	21.1	*HMGB1, NEIL1, NEIL3, MUTYH, UNG, LIG1*
nucleosome assembly	7.5	*H2B1, HIST1H2BJ, HMGB1, HIST1H2BI, HIST1H1C, DCK, HIST1H1A, HIST1H1E, HIST1H1D*
Up-regulated genes in DC**		
Cholesterol import	>100	*APOA1, CD36*
Cholesterol transport	95.5	*APOB, APOA1, CD36*
Sterol transport	90.9	*APOB, APOA1, CD36*
Sterol import	>100	*APOA1, CD36*
Triglyceride metabolic process	93.2	*APOB, APOA1, AGPAT9*

*Distal jejunum.

**Descending colon.

***Number of genes observed in the uploaded list over the expected number genes involved in that term for *Bos taurus* (which is determined by the Gene Ontology database).

**Table 2:** Differentially expressed genes in distal jejunum enriched for GO terms that are associated with immune functions.

Gene	Log2 (fold change)	Location	Type(s)
*CCL2*	1.1	Extracellular Space	cytokine
*CXCL10*	2.5	Extracellular Space	cytokine
*CXCL9*	3.2	Extracellular Space	cytokine
*FASLG*	1.4	Extracellular Space	cytokine
*IL1A*	1.6	Extracellular Space	cytokine
*TNFSF10*	1.2	Extracellular Space	cytokine
*CD274*	2.1	Plasma Membrane	enzyme
*GBP5*	2.3	Plasma Membrane	enzyme
*IDO1*	2.9	Cytoplasm	enzyme
*CCR9*	1.2	Plasma Membrane	G-protein coupled receptor
*GPR171*	1	Plasma Membrane	G-protein coupled receptor
*GPR55*	1.3	Plasma Membrane	G-protein coupled receptor
*ITK*	1.1	Cytoplasm	kinase
*BATF2*	2	Other	other
*CLNK*	1.4	Cytoplasm	other
*LCP2*	1.1	Cytoplasm	other
*THEMIS*	1.4	Cytoplasm	other
*PTPN22*	1.2	Cytoplasm	phosphatase
*CD6*	1.1	Plasma Membrane	transmembrane receptor
*IL18R1*	1.3	Plasma Membrane	transmembrane receptor
*IL1RL1*	1.4	Plasma Membrane	transmembrane receptor
*KLRK1*	1.3	Plasma Membrane	transmembrane receptor

**Table 3:** IPA downstream functional analysis and pathways analysis for DE genes between non-shedders and super-shedders in distal jejunum, cecum, and descending colon, no functional terms or pathways with |z| ≥ 2 were enriched for the other tissues.

Tissues	Functional terms/pathways	z-score	Involved genes
Distal jejunum	leukocyte migration	2.2	*CCL2, CCR9, F3, GPR132, S1PR2*
Distal jejunum	LXR/RXR activation	−2.4	*APOE, ARG2, CCL2, IL1A, IL1RL1, PTGS2*
Distal jejunum	B-cell receptor signaling	−2.0	*BCL6, BLNK, CD79A, CD79B, EBF1*
Descending colon	proliferation of T-cells	2.0	*APOA1, CD36, GP2, GPAM*

In addition to immune functions, IPA pathway analysis of DE genes in distal jejunum showed inhibited LXR/RXR activation pathway (z-score ≤ −2.0) which is associated with increased cholesterol absorption in SS. Six genes were associated with this pathway, and two genes (*APOE, ARG2)* were down-regulated, while four genes (*CCL2*, *IL1A, IL1RL1, PTGS2*) were up-regulated (Table 3). Similarly, for the DE genes in the descending colon, five enriched GO terms were also associated with cholesterol transportation, including “cholesterol import”, “sterol import” and “cholesterol transport” (Table 1). Four DE genes (*APOB*, *APOA1*, *CD36* and *AGPAT9*) associated with these GO terms were up-regulated (Table 1).

### SNP identification of DE genes

**Figure 5:**
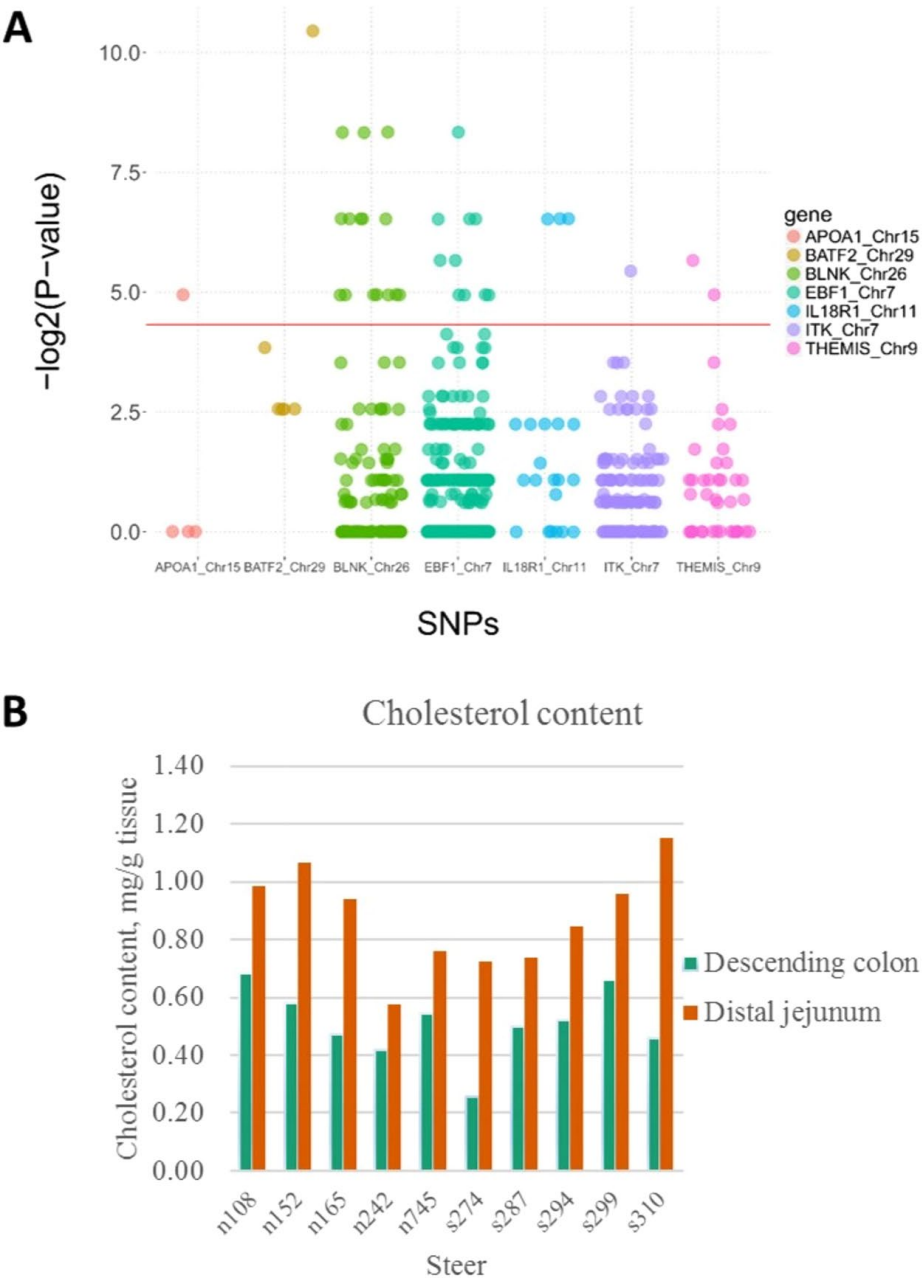
(**A**) SNPs detected from RNA-Seq data. The x-axis shows the location of a SNP on the genes, and y-axis shows the −log2(p-values). The SNPs that shows association with super-shedding phenomena are presented above the red line (p-value < 0.05). (**B**) Cholesterol quantification results.

To identify the factors that regulate the observed varied expression between SS and NS, 33 DE genes associated with immune functions and cholesterol transportation (Supplementary Table S3) were then subjected to SNP identification analysis. In total, 1,575 SNPs were identified based on RNA-Seq dataset of these DE genes, and 1,477 of them have been previously reported in the dbSNP database^32^. Among those SNPs, 67 were three prime UTR variants, 9 were five prime UTR variants, 1,283 were intronic variants, 41 were missense variants, 9 were splice region variants (3 missenses variants), and 71 were synonymous variants. The association analysis (Fisher’s exact test) showed that 33 SNPs (Table 4) in seven genes (p-value < 0.05) including *BATF2*, *THEMIS*, *ITK*, *BLNK*, *IL18R1, EBF1*, and *APOA1* were associated with super-shedding (Fig. 5A). As indicated by GO term enrichment and IPA pathway analysis, *BATF2*, *THEMIS*, *ITK*, *BLNK*, *IL18R1* and *EBF1* were related to leukocyte activation and *APOA1* was associated with cholesterol transportation. Of these 33 SNPs, rs42084078 in *BLNK* and rs109755291 in *IL18R1* were missense variants and rs384985356 in *APOA1* was a synonymous variant, while the others were intronic variants. Furthermore, rs42084078 (A/G) causes an amino acid difference (A/V) at position 101 of BLNK (Accession number: NP_001039519.1), and rs109755291 (C/T) also causes an A/V difference at position 531 of IL18R1 (Accession number: XP_005212515.1).

**Table 4:** SNPs which showed association with super-shedding phenomena.

Gene	Variant.ID	Location	Alleles	Freq_NS*	Freq_SS**	Consequence	p-value
APOA1	rs384985356	15:27932563	A/C	A, 50%	A, 100%	Synonymous variant	0.033
BATF2	rs42191303	29:43828428	C/T	C, 100%	C, 20%	Intron variant	0.001
BLNK	rs42088770	26:17382354	A/T	A, 100%	A, 50%	Intron variant	0.033
BLNK	rs110491800	26:17395942	A/G	A, 50%	A, 100%	Intron variant	0.033
BLNK	rs110241837	26:17398172	G/T	G, 0%	G, 70%	Intron variant	0.003
BLNK	rs109825977	26:17398543	A/G	A, 0%	A, 60%	Intron variant	0.011
BLNK	rs209836657	26:17398630	G/C	G, 0%	G, 70%	Intron variant	0.003
BLNK	rs42088798	26:17399306	G/C	G, 0%	G, 70%	Intron variant	0.003
BLNK	rs385916156	26:17399758	T/A	T, 0%	T, 60%	Intron variant	0.011
BLNK	rs211039227	26:17401401	T/A	T, 0%	T, 60%	Intron variant	0.011
BLNK	rs42088814	26:17401969	A/G	A, 100%	A, 40%	Intron variant	0.011
BLNK	rs42088815	26:17402059	T/C	T, 100%	T, 50%	Intron variant	0.033
BLNK	rs42084078	26:17419883	A/G	A, 0%	A, 50%	Missense variant	0.033
BLNK	rs135453434	26:17427529	C/T	C, 100%	C, 50%	Intron variant	0.033
BLNK	rs42084065	26:17428014	T/C	T, 100%	T, 50%	Intron variant	0.033
BLNK	rs132877528	26:17431964	C/T	C, 0%	C, 50%	Intron variant	0.033
BLNK	rs134560601	26:17432163	A/G	A, 0%	A, 60%	Intron variant	0.011
IL18R1	rs109078612	11:7153863	T/C	T, 0%	T, 60%	Intron variant	0.011
IL18R1	rs109700098	11:7169952	T/C	T, 0%	T, 60%	Intron variant	0.011
IL18R1	rs109755291	11:7179544	C/T	C, 0%	C, 60%	Missense variant	0.011
ITK	rs109137099	7:70923893	T/C	T, 80%	T, 20%	Intron variant	0.023
THEMIS	rs135858596	9:66782892	C/A	C, 30%	C, 90%	Intron variant	0.020
THEMIS	rs207702093	9:66756813	C/T	C, 100%	C, 50%	Intron variant	0.033
EBF1	rs207972417	7:72475725	C/T	C, 100%	C, 40%	Intron variant	0.011
EBF1	rs207622846	7:72477936	T/C	T, 100%	T, 50%	Intron variant	0.033
EBF1	rs209147903	7:72478125	G/A	G, 100%	G, 40%	Intron variant	0.011
EBF1	rs211282271	7:72478236	T/C	T, 100%	T, 50%	Intron variant	0.033
EBF1	rs384133035	7:72481066	A/G	A, 100%	A, 50%	Intron variant	0.033
EBF1	rs41656033	7:72582112	G/C	G, 100%	G, 30%	Intron variant	0.003
EBF1	rs132998336	7:72588676	G/A	G, 90%	G, 30%	Intron variant	0.020
EBF1	rs135578882	7:72616057	C/T	C, 100%	C, 40%	Intron variant	0.011
EBF1	rs43524161	7:72701165	C/G	C, 50%	C, 0%	Intron variant	0.033
EBF1	rs29027619	7:72710782	G/C	G, 70%	G, 10%	Intron variant	0.020

*Allele frequency in non-shedders, total frequency of two alleles are 100%.

**Allele frequency in super-shedders, total frequency of two alleles are 100%.

### Cholesterol quantitation in distal jejunum and descending colon

To further verify the identified potential cholesterol metabolism difference in distal jejunum and in descending colon between SS and NS, the cholesterol concentrations in these two tissues were evaluated. The cholesterol content ranged from 0.57 to 1.15 mg/g in the distal jejunum, and 0.26 to 0.68 mg/g in the descending colon. The cholesterol content was higher in small intestinal tissue than in large intestinal tissue (t-test, p-value < 0.05); however, there was no statistical difference between SS and NS in both tissues (p-values were 0.89 and 0.48 for descending colon and distal jejunum, respectively) (Fig. 5B).

## Discussion

To our knowledge, this study is the first to investigate gene expression (transcriptome) throughout the whole gastrointestinal tract of beef cattle with different *E. coli* O157 shedding phenotypes. Although the rectal-anal junction has been suggested to be the primary *E. coli* O157 colonisation site, this pathogen has also been isolated from other regions of the digestive tract^33^, suggesting that the environment throughout the gastrointestinal tract could impact on the colonisation of this pathogen which can attribute to the fecal shedding capacity of the animals. Therefore, the information obtained from the genome wide transcriptome can help us understand gut physiology and biology of bovine intestinal tract at a molecular level and how it may differ between SS and NS, which could partially explain the super-shedding phenomenon. To better assess such information, the whole intestinal tissue (epithelium and tissues underneath) for each region was used for transcriptome profiling as the tissues underlying the epithelium (such as the lymphoid structures) may also play a role in super-shedding phenomena^16^.

Similar numbers of expressed genes were detected in all gut tissues, which was not surprising due to their physiological nature as the digestive tract. When the whole transcriptomes were compared, most of tissues (especially small intestinal tissues) showed no segregation between NS and SS, suggesting that the variation in *E. coli* O157 shedding status was not the key factor to influence the overall host gut transcriptomes. Indeed, these animals were healthy and no significant difference was observed in terms of their growth. The association of immune functions with small intestine regions, especially for distal jejunum, was in agreement with a previous report that jejunum also plays a role in mucosal immunity because Peyer’s patches (PP) are present in this region^34^, and ileal PP extend from the distal jejunum to ileocecal valve^34^. Higher level of activity of immune functions in the small intestine over the large intestine in beef steers suggests potential lower chance for *E. coli* O157 to colonize in small intestine. Thus, the colonisation of *E. coli* O157 in large intestine of cattle, especially in the RAJ^16^, may be a result from lower host immune activity of these regions. Further validation is needed to measure whether lymphoid structures in small intestine can contribute to the tropism of *E. coli* O157. The separation of transcriptome in cecum and descending colon between NS and SS further suggests that difference in hindgut physiological environment regulated by gene expression profiles may contribute to the difference in *E. coli* O157 shedding. Such findings were also in agreement with previous report that the colonisation of *E. coli* O157 was prevalent in lower digestive tracts of challenged yearling cattle, including cecum and colon^35^. It is possible that the environment of hindgut of SS is more favourable for *E. coli* O157 to survive, proliferate and colonise. Future studies to quantify the *E. coli* O157 population and measure the physiological parameters as well as immunological features in each region of the gut is necessary to identify alternation in which host functions through the gut can regulate *E. coli* O157 colonisation and the high level faecal shedding of *E. coli* O157.

**Figure 6:**
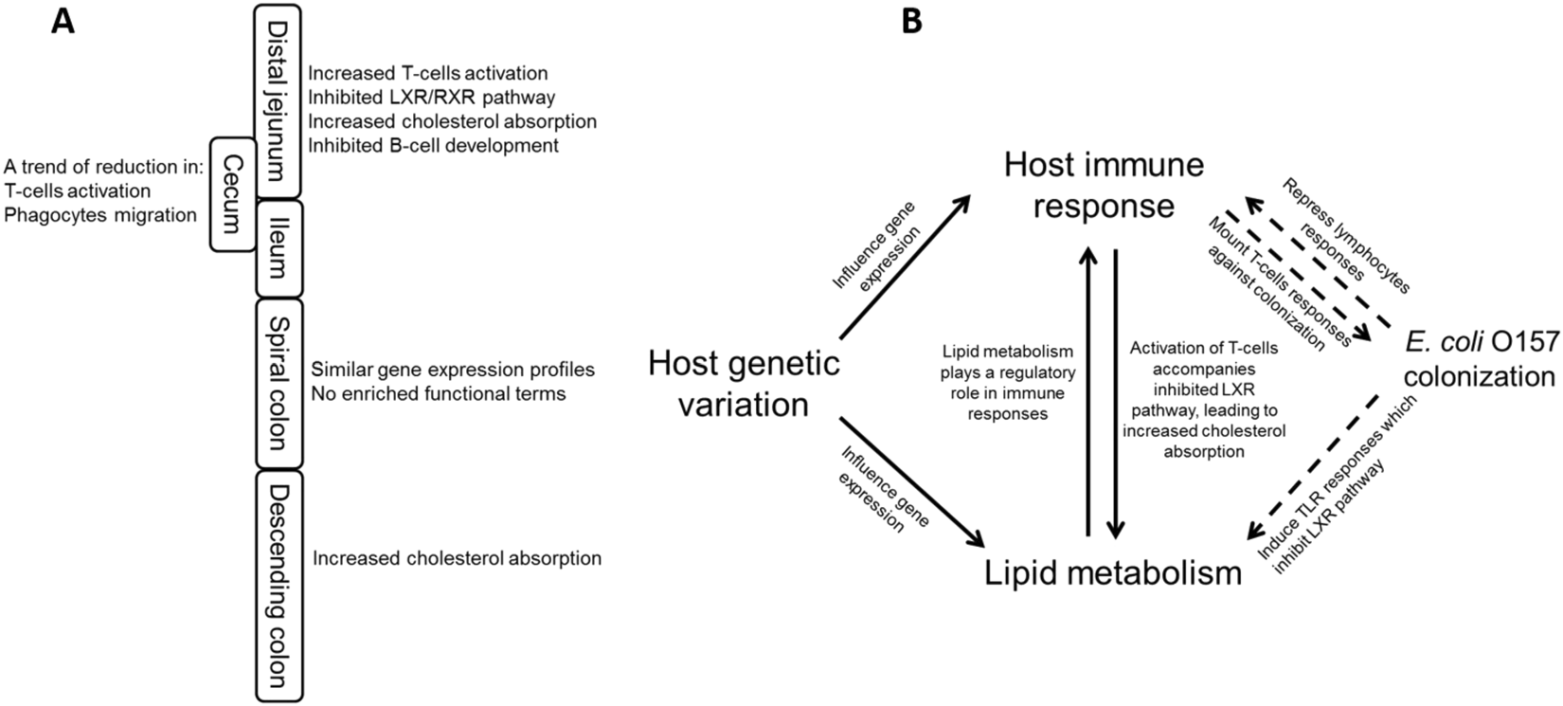
Possible host and *E. coli* O157 interactions in super-shedder cattle. (**A**) Changes in functions of different intestinal regions of super-shedders. (**B**) Proposed potential host mechanism involved in lipid metabolism, host immune responses that could be associated with *E. coli* O157 colonisation in the gut of cattle.

Although there were no significant differences in core transcriptomes among the small intestinal regions (indicated by PCA analysis), DE genes were detected for each gut region between SS and NS. In the small intestinal region, lower number of DE genes were identified in duodenum and proximal jejunum compared with distal jejunum, suggesting that the gut environment in anterior part of small intestine is similar between SS and NS. The highest number of DE genes (248, including 181 down-regulated and 67 up-regulated) was detected in distal jejunum with the up-regulated DE genes *F3*, *GPR132*, *CCR9*, *CXCL9* and *CXCL10* involved in several GO terms including “cytokine-mediated signaling pathway”, “positive regulation of intracellular signal transduction”, “defense response”. Previous findings have revealed that these genes are directly involved in T-cell related functions. The *F3* (also called TF, log2 fold change: 1.3) product was reported to play a regulatory role in recruiting leukocytes in intestine of mice^36^, whereas the *GPR132* (also called *G2A*, log2 fold change: 1.3) protein was reported to enhance chemotaxis of T-cells *in vitro* ^37^. Similarly, the requirement of *CCR9* (log2 fold change: 1.2) for migration of T helper 17 cells (Th17) to the small intestine was demonstrated using mice^38^, and CXCL9 (log2 fold change: 3.2) and CXCL10 (log2 fold change: 2.5) can induce chemotaxis of T-helper cells^39^. In addition, the cytokine *IL1A* and two cell-membrane receptor genes, *IL18R1* and *IL1RL1* were upregulated and were involved in GO terms including “cytokine-mediated signaling pathway” and “leukocyte activation” in distal jejunum SS. *IL1A* (log2 fold change: 1.6) protein plays a key role in the differentiation of Th17 cells^40^; IL18R1 (log2 fold change: 1.3) was suggested to promote cell-mediated responses^41^; and IL1RL1 (log2 fold change: 1.4) is involved in activation of T-helper cells^41, 42^. Up-regulation of these genes suggests a potential increase in T-cell migration, differentiation and proliferation in the distal jejunum of SS (Fig. 6A).

For descending colon, the functional analysis of the up-regulated DE genes also suggests a trend of increased proliferation of T-cell (z-score ≥ 2.0) in SS. The up-regulation of *CD36* (log2 fold change: 4.1) was reported to increase T-cell proliferation i*n vitro* ^43^; *GPAM*, also known as *GPAT-1*, (log2 fold change: 2.4) was reported to increase T-cell proliferation in mice^44^. These findings are in agreement with previous findings that increased expression of genes involved in the proliferation of T-cells in the rectum of *E. coli* O157 challenged cattle^45^. However, different from our previous transcriptomic analysis of rectal tissues which suggested a potential decrease in both humoral and cell-mediated immune functions in SS^11^, our current study indicates potentially enhanced T-cell migration and proliferation at the distal jejunum and descending colon of SS. In the gut associated lymphoid system, T-cells need to migrate from thymus to the gut epithelium to become intraepithelial T-cells (IETs), which are mature and activated cells serve as front line to protect the intestinal epithelium^46^. Compared with NS, the gut transcriptomes of SS suggest a higher level of T-cell migration in locations anterior to RAJ, while lower T-cell migration and quantity at RAJ^11^, suggesting a potential dysregulation of migration of T-cells in SS cattle which may result in reduced IETs accumulation at RAJ, leaving it more vulnerable to *E. coli* O157 colonisation.

In addition, the functional analysis of down-regulated DE genes in distal jejunum suggest association between genes including *CD79A*, *CD79B*, *BLNK*, *BCL6* and *EBF1* and decreased B-cell signalling. CD79A (log2 fold change: −2.2) and CD79B (log2 fold change: −4.3) are components of B-cell receptor (BCR) that initiates the B-cell signalling pathway^47^. Upon BCR interaction with antigens, BLNK (log2 fold change: −2.5) can be phosphorylated, leading to downstream signal transduction^48^, such as the mediation of transcription by BCL6 and EBF1, two transcription factors essential to B-cell maturation^49, 50^. *E. coli* O157 mediated activities, such as Shiga-toxin production, were reported to suppress lymphocyte responses in cattle^51^, which may partially explain why the transcriptome analysis suggested inhibited B-cell signalling pathway in distal jejunum and the trend of reduction in lymphocyte activation and migration in cecum of SS.

Besides the potential involvement of immune functions, the pathway analysis and GO term enrichment of DE revealed inhibited LXR/RXR pathway and altered cholesterol transportation in distal jejunum and descending colon of SS. It has been reported that activation of LXR/RXR pathway in intestinal tract leads to decreased cholesterol absorption/synthesis and increased excretion of cholesterol in faeces^52^. Thus, inhibition of LXR/RXR pathway could lead to increased cholesterol absorption in the distal jejunum of SS. In addition, the up-regulated DE genes in the descending colon of SS, including *APOB* (log2 fold change: 8.9), *APOA1* (log2 fold change: 6.0), *CD36* (log2 fold change: 4.1) and *AGPAT9* (log2 fold change: 3.2) also suggest altered cholesterol transportation in the SS. *APOB* encodes a key component of chylomicrons and low density lipoproteins which are responsible for cholesterol transportation between luminal content and intestinal tissues^53^. APOA1 was reported to regulate cholesterol transportation between cell membrane^54^ and CD36 can facilitate cholesterol absorption in the intestinal tract^55^. Moreover, AGPAT9 was reported to be involved in cholesterol metabolism, because the deficiency of AGPAT9 (in mice) led to dysregulation in cholesterol metabolism with higher free cholesterol and cholesteryl esters in plasma and liver^56^. Our observations that differences in the expression of genes involved in cholesterol transportation and immune functions in the gut between SS and NS are not likely due to coincidence, as lipid metabolism plays a key role in immune system^57^. The LXR pathway was reported to interact with both the innate and adaptive immune systems, especially with macrophage functions^58, 59^. In addition, Bensinger *et al*. reported that increased T-cell activation in mice was accompanied by inhibition of LXR pathway and promotion of cholesterol synthesis^58^. A possible mechanism is that cholesterol is required for cell membrane construction and cell proliferation, and thus increased sterol absorption/synthesis may act as a factor to lymphocytes proliferation^58^. Similar in the report by Bensinger *et al*.^58^, current findings also indicated potentially increased T-cell migration and proliferation accompanied by a trend of increased lipid absorption in the distal jejunum and descending colon of SS, suggesting that both alteration of host immune functions and lipid metabolism could influence *E*. *coli* O157 shedding. However, the increased absorption of cholesterol in the SS may have compensated the potentially increased utilization of cholesterol for T-cell proliferation in epithelial tissues of the distal jejunum and descending colon, which may explain why no differences were observed for cholesterol concentrations in distal jejunum and descending colon. Also, cholesterol may have been transported to the liver via mesenteric lymph upon absorption, as intestine is not an organ of cholesterol storage. In addition, post-transcriptional regulation may also influence the expression of proteins which are the major players in physiological functions. Therefore, although the gene expression suggested the increased cholesterol absorption, the measurable cholesterol level in the distal jejunum and descending colon have remained unchanged, due to immediate utilization, transportation or/and post-transcriptional regulations. Further validation is required to investigate the transcriptome of the liver, and the cholesterol levels in the liver and blood, as well as the post-transcriptional regulations in the intestine of NS and SS.

It is noticeable that highly upregulated expression of *FOLH1* (log2 change fold: 4.5 in RNA-Seq results; for qPCR measurement, the ΔCq was −1.04 in NS and −1.97 in SS, ΔCq was calculated by Cq_FOLH1_ - Cq_β-actin_, lower ΔCq indicated higher expression) occurred in descending colon of SS, suggesting that the expression level of *FOLH1* was up-regulated in SS. Up-regulation of *FOLH1* was observed in intestine of rat in a folate-deficient situation^60^. Because the environmental and dietary factors are the same in the NS and SS, it is possible that the potential folate-deficiency in descending colon was due to lower density of folic acid synthesizing commensal bacteria, including beneficial microorganisms such as *Bifidobacterium* species^61^. To identify how host-commensal interactions can influence *E. coli* O157 shedding requires further research, such as 16S rDNA amplicon sequencing for mucosa attached microbes and metagenomics studies.

Further SNPs discovery analysis identified 33 SNPs in seven DE genes associated with immune functions and cholesterol transportation. Among those SNPs, rs42084078 (in *BLNK*) and rs109755291 (in *IL18R1*) are non-synonymous, and allele A of rs42084078 and allele C of rs109755291 were only seen in SS. Thirty of the SNPs showing association with super-shedding were in the intron of genes *BATF2*, *BLNK*, *IL18R1*, *ITK*, *THEMIS* and *EBF1*. Although SNPs in the intron do not alter protein sequences, they are still important since introns are known to regulate gene expression, and intronic variation was reported to be associated with phenotypes^62^. For example, the DE gene *EBF1* is a transcription factor essential for B-cell development from innate lymphoid cells^39^, and intronic SNPs in *EBF1* was reported to be associated with autoimmune disease in human^63^. Therefore, we speculate that the detected genetic variations may influence their expression and lead to difference in B-cell signalling, T-cell responses and cholesterol absorption in the gastrointestinal tract between SS and NS. Future validation study is needed for the identified SNPs, and current results can be used as a reference for selection of candidate gene/SNPs for testing using larger populations as well as with more defined shedding status through a long-term monitoring approach.

It is known that shedding levels vary greatly in SS, ranging from 10^4^ to 10^9^ CFU/g of faeces^5^, with three types of SS being observed: non-persistent shedders (shedding <14 days), moderately persistent shedders (shedding ~30 days), and persistent shedders (shedding last several months)^4^. As described by Munns (2015)^22^, the SS used in this study had different faecal *E. coli* O157 counts before and upon their slaughter. For example, SS 310 was slaughtered on the 5th day of shedding and the faecal *E. coli* O157 in this animal upon slaughter was still greater than 10^4^ CFU/g of faeces. Similarly, SS 274/SS 287 and SS 294/SS 299 were slaughtered on the 8th day and 11th day, respectively, and their shedding level was below 10^4^ CFU/g of faeces at the slaughter. Different shedding patterns among these five SS may be the reason underlying the variation observed in gene expression through the gut of SS. However, since they were monitored for only 11 days at most, it is difficult to define the which types of shedder they belonged to. Therefore, future studies using the samples collected from long term monitored SS are needed to determine how the gut transcriptomes differ among different types of SS.

## Conclusion

Our results suggest that (1) the bovine small intestine may have a more active immune system than the large intestine, making the distal colon towards rectum more prone to colonisation with *E. coli* O157; (2) the distal jejunum and descending colon of SS showed potentially higher levels of T-cell migration and proliferation, and cholesterol absorption, as well as inhibited B-cell maturation (Fig. 6A); (3) host genetic variation may be one of the mechanisms to affect the expression of genes involved in immune function and cholesterol absorption that may play a role in *E. coli* O157 shedding in SS, but genetic association analysis with larger number of animals is required to validate such speculation. Based on the transcriptome data, we speculate that host mechanisms, including genetic variation, alternation in immune functions and cholesterol metabolism, may be critical to super-shedding (Fig. 6B). Although this study is stronger at revealing the alteration of gene expression during high level shedding of *E. coli* O157 in cattle, potential host functions that could influence SS shedding were also identified. Further research is needed focusing on the associations among host mechanisms (such as genetics, immunity and cholesterol metabolism, as well as host-microbial interaction) and the *E. coli* O157 immunomodulatory effects, with larger populations of cattle and/or using a longitude approach. In addition, long-term monitoring faecal *E. coli* O157 for animals used in future studies to define NS as negative control and the type of SS (temporary, intermediate, and persistent) is critical to investigate more specific host effects that are associated with super-shedding.

### Importance

*Escherichia coli* O157:H7 (*E. coli* O157) is a foodborne pathogen which causes human illness as well as significant economic loss for food industry. Cattle are the primary reservoir for *E. coli* O157, and some animals can shed greater than 10^4^ CFU/g faeces, who are considered as super shedders. To date, the host mechanisms involved in *Escherichia coli* O157 super-shedding in cattle is largely unknown. This study performed genome wide transcriptome analysis of the whole gastrointestinal tract of super-shedders in comparison with non-shedders and have revealed genes involved in immunity and cholesterol metabolism significantly differed between the two groups of animals and identified SNPs in seven of these DE genes. The identified SNPs could serve as potential genetic markers to select and breed cattle with the “suitable” gut environment allowing no or less *E. coli* O157 shedding in cattle. These findings provide valuable information in understanding of host-microbial interactions in super-shedding phenomena.

## Electronic supplementary material

Supplementary figures

Supplementary Table S1

Supplementary Table S2

Supplementary Table S3
