# Supplementary material for: Host mechanisms involved in cattle Escherichia coli O157 shedding: a fundamental understanding for reducing foodborne pathogen in food animal production
Source: Sci Rep. 2017 Aug 9;7:7630. doi: 10.1038/s41598-017-06737-4 (PMC5550497; doi:10.1038/s41598-017-06737-4)
Supplement: Supplementary file 1 — Supplementary figures [file 41598_2017_6737_MOESM1_ESM.pdf]

Supplementary Figures

Host mechanisms involved in cattle *Escherichia coli* O157 shedding: a fundamental understanding for reducing foodborne pathogen in food animal production

Ou Wang,[1] Tim A. McAllister,[2] Graham Plastow,[1] Kim Stanford,[3] Brent Selinger,[4] and Le Luo Guan[1*]

[1]Department of Agricultural, Food and Nutritional Science, University of Alberta, Edmonton, AB, Canada, T6G 2P5; [2]Agriculture and Agri-Food Canada, Lethbridge Research Centre, Lethbridge, AB, Canada, T1J 4B1; [3]Alberta Agriculture and Forestry, Lethbridge, AB, Canada, T1J 4V6; and [4]Department of Biological Sciences, University of Lethbridge, Lethbridge, AB, Canada, T1K 3M4

*Corresponding author.

Contact information: 410 Agriculture/Forestry Centre, University of Alberta, Edmonton, AB, Canada T6G2P5; Tel, 780-492-2480; Fax, 780-492-4265; E-mail. lguan@ualberta.ca

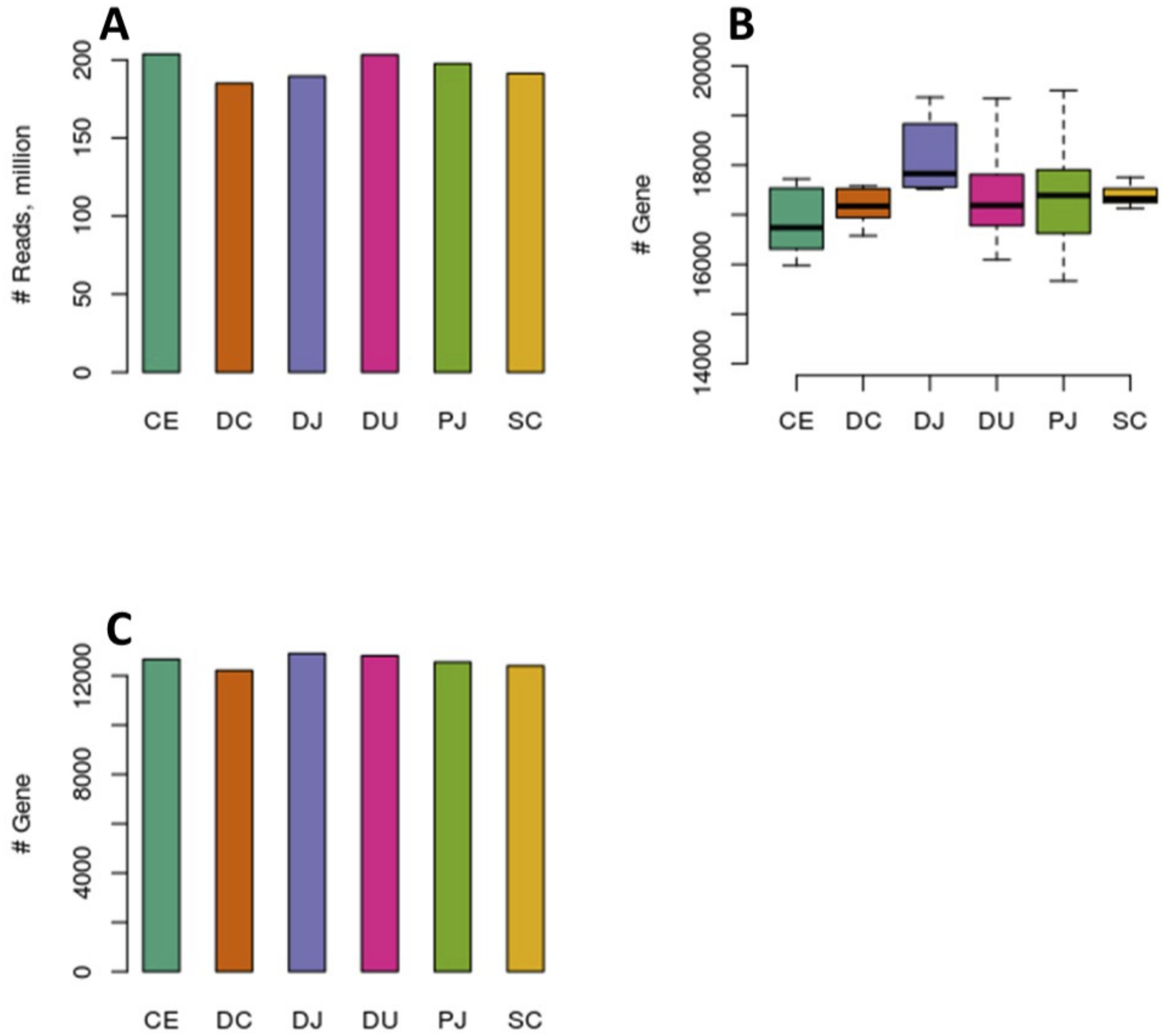

Figure S1. RNA-Sequencing results: (A) Number of reads generated for all tissue samples, ranged from 185 million to 203.7 million. (B) Number of genes detected in each sample, ranged from 16,846 ± 639 (CE) to 18,137 ± 696 (DJ). (C) Number of genes in core transcriptome of each gene, ranged from 12,216 (in DC) to 12,905 (in DJ). DU, duodenum; PJ, proximal jejunum; DJ, distal jejunum; CE, cecum; SC, spiral colon; DC, descending colon.

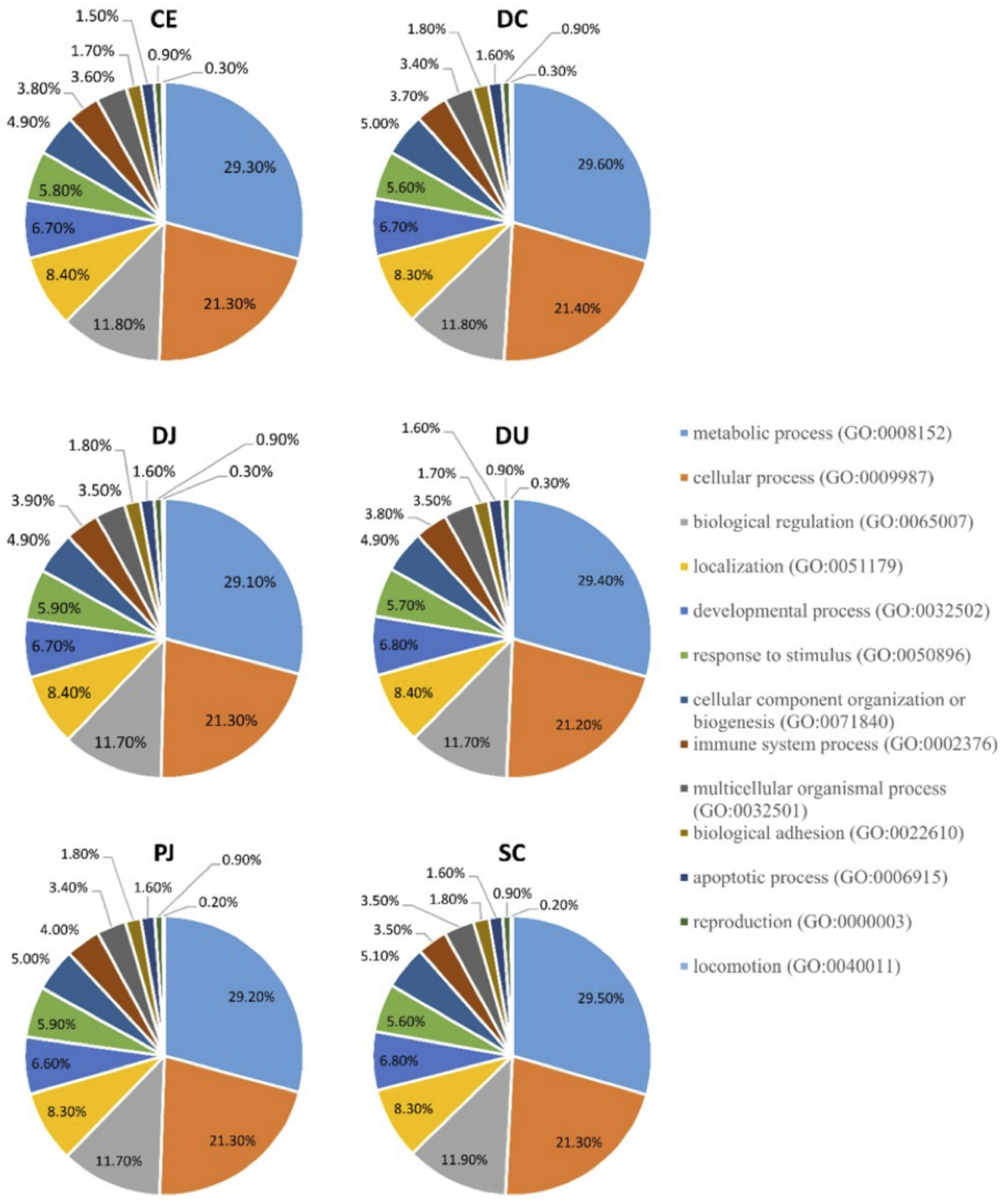

Figure S2. Top GO terms enriched for core transcriptome of each intestinal region. The pie charts indicated the percentage of "number of genes / number of GO term hits" scale. About 50% of genes are associated with two GO terms including "metabolic process", "cellular process". DU, duodenum; PJ, proximal jejunum; DJ, distal jejunum; CE, cecum; SC, spiral colon; DC, descending colon.

B Cell Receptor Signaling

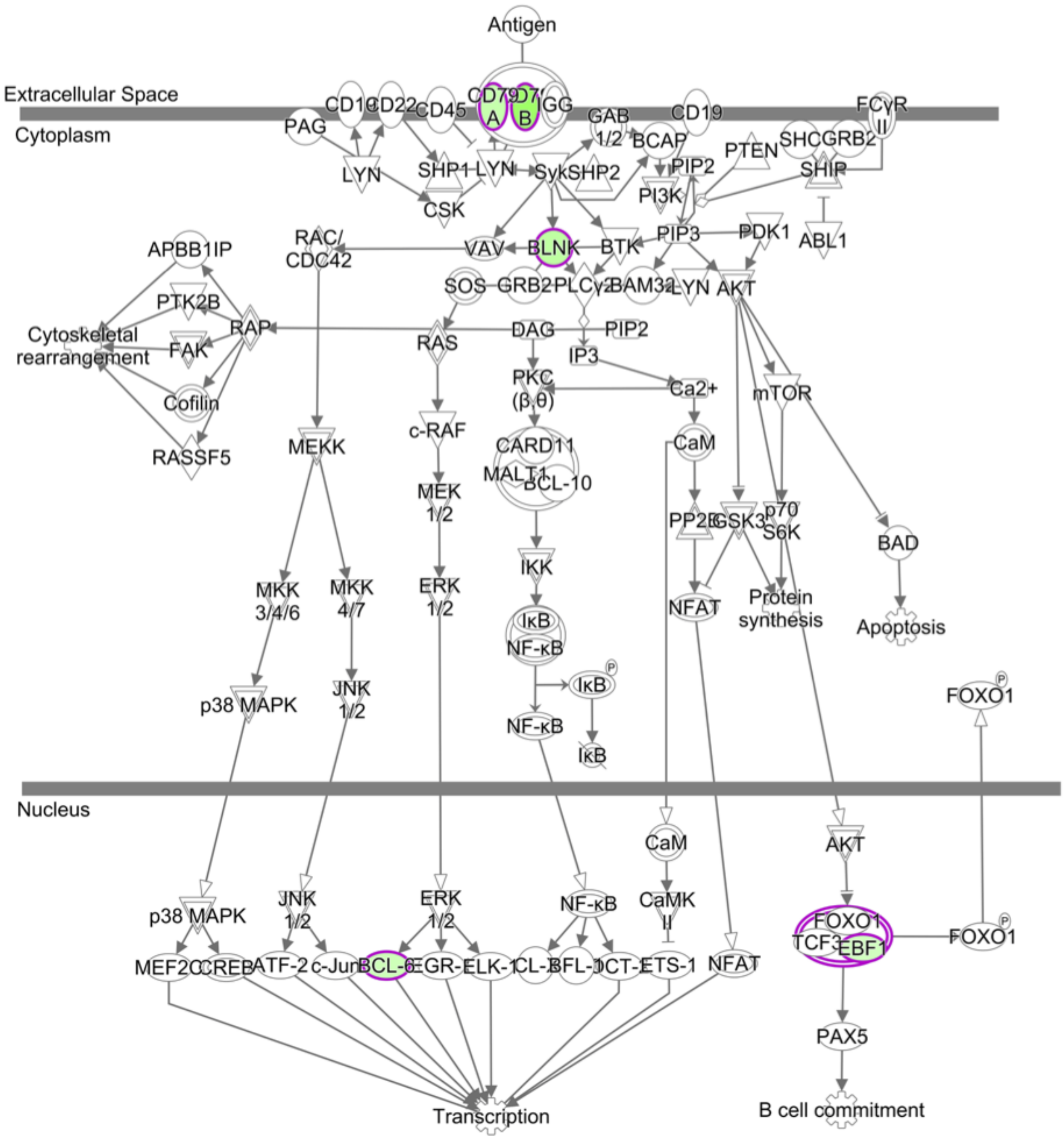

© 2000-2016 QIAGEN. All rights reserved.

Figure S3. B-cell signalling pathway, the green coloured genes were down-regulated in the distal jejunum of super-shedders. The figure was generated using Ingenuity Pathway Analysis (IPA, QIAGEN, Redwood City, CA, United States www.qiagen.com/ingenuity). Permission was granted by QIAGEN Silicon Valley to use the copyrighted figure for publication. Figures produced from IPA are available under an open-access CC-BY license for purposes of publication.
